# Comparative Evaluation of Antagonistic Tooth Wear After Placement of Crowns Made From Three Different Materials Using a Digital Intraoral Scanner: An In Vivo Study

**DOI:** 10.7759/cureus.113603

**Published:** 2026-07-29

**Authors:** Ubaid Iqbal Shah, Adiba Ihsan, Shazia Kosar, Schubert Anto R., Mansi Manohar Gadhe, Prabhjeet Kaur

**Affiliations:** 1 Department of Prosthodontics and Crown and Bridge, Government Dental College and Hospital, Srinagar, IND; 2 Department of Oral Medicine and Radiology, Kothiwal Dental College and Research Centre, Moradabad, IND

**Keywords:** antagonistic tooth wear, dental crowns, digital intraoral scanning, direct metal laser sintering, peek, zirconia

## Abstract

Introduction

Antagonistic tooth wear is a significant concern in fixed prosthodontics as it can compromise long-term occlusal stability and natural tooth integrity. Advances in restorative materials have introduced options with varying wear behaviors; however, direct clinical comparisons using digital technologies remain limited. This in vivo study aimed to comparatively evaluate antagonistic tooth wear produced by crowns fabricated by direct metal laser sintering (DMLS), rubellite-layered zirconia, and polyetheretherketone (PEEK) materials using digital intraoral scanning over 12 months.

Materials and methods

A total of 36 patients (18 males and 18 females; mean age: 30.27 ± 3.45 years) fulfilling the inclusion criteria were enrolled and assigned to three equal groups of 12 patients each. Each group comprised six males and six females. The crowns were fabricated using a fully digital workflow and cemented. Antagonist tooth wear on the opposing mandibular first molars was assessed by superimposing baseline, 6-month, and 12-month digital intraoral scans using dedicated computer-aided design (CAD) software. The linear changes were quantified. Data were analyzed using two-way mixed-design analysis of variance (ANOVA), followed by Tukey’s post hoc test.

Results

All the groups showed a statistically significant increase in antagonist tooth wear between 6 and 12 months. DMLS crowns produced the highest wear at both intervals (6 months: 0.0787 ± 0.0176 mm; 12 months: 0.1018 ± 0.0326 mm), followed by rubellite-layered zirconia crowns (0.0450 ± 0.0238 mm and 0.0605 ± 0.0249 mm). PEEK crowns demonstrated the lowest wear values (0.0288 ± 0.0123 mm at 6 months and 0.0433 ± 0.0129 mm at 12 months). The main effect of material type was highly significant, with DMLS differing markedly from the other two materials, whereas the difference between zirconia and PEEK was not statistically significant.

Conclusion

Among the study parameters, PEEK crowns exhibited the most favorable wear behavior against natural enamel, followed by rubellite-layered zirconia crowns. Digital scanning has proven to be effective for precise wear quantification. These findings support material-specific selection to minimize iatrogenic enamel loss, while maintaining esthetics and function.

## Introduction

Prosthodontics focuses on restoring missing or damaged teeth and their associated structures to re-establish function, esthetics, comfort, and oral health. Among the fixed prosthetic options, dental crowns are the most commonly prescribed restorations for teeth compromised by extensive caries, trauma, endodontic treatment, or pathological wear. Advances in materials science and digital technologies have expanded the range of crown materials, enabling clinicians to choose restorations based on their mechanical properties, esthetics, biocompatibility, and wear behavior [1,2]. However, the interaction between restorative materials and opposing natural dentition remains a critical factor in long-term clinical success. Antagonistic tooth wear, the progressive loss of enamel structure on the opposite tooth due to contact with restorative materials, is a significant clinical concern, as excessive wear can lead to occlusal instability, reduced vertical dimension, dentin hypersensitivity, and temporomandibular joint disorders [3,4].

Tooth wear is an irreversible process that is primarily driven by attrition (tooth-to-tooth contact during mastication or parafunction). The extent of antagonist wear depends on occlusal load, contact duration, material hardness, surface roughness, elastic modulus, and microstructure. Traditional restorative materials, such as ceramics, can be abrasive because of their higher hardness compared to enamel, while metallic and polymer-based materials often exhibit better wear compatibility [5]. Direct metal laser sintering (DMLS) produces cobalt-chromium crowns with excellent strength and marginal fit; however, porcelain veneering can increase the abrasiveness [6]. Rubellite-layered zirconia offers superior fracture toughness and aesthetics through its zirconia core and specialized veneering porcelain, with surface characteristics that potentially improve the wear behavior over conventional layered zirconia [7]. Polyetheretherketone (PEEK) is a high-performance thermoplastic polymer that provides a low elastic modulus close to that of dentin, shock-absorbing properties, and minimal abrasiveness, making it promising for patients at risk of tooth wear [8].

Conventional methods for assessing tooth wear (clinical indices, photographs, or stone casts) are subjective and lack precision in detecting small volumetric changes [9]. Digital intraoral scanners now enable accurate, non-invasive quantification of linear and volumetric wear through the superimposition of serial scans using software such as Exocad [10]. This technology overcomes the limitations of traditional approaches and supports reliable longitudinal evaluation in clinical settings. Although numerous in vitro studies have been conducted, they often fail to replicate intraoral conditions involving saliva, temperature fluctuations, and individual occlusal patterns [5,11]. There is a notable lack of in vivo clinical studies that directly compare antagonistic tooth wear produced by DMLS, rubellite-layered zirconia, and PEEK crowns using digital scanning techniques.

The aim of the present study was to comparatively evaluate antagonistic tooth wear produced by crowns fabricated from DMLS, rubellite-layered zirconia, and PEEK materials using a digital intraoral scanner in an in vivo clinical setting. The objectives were to fabricate and place full-coverage crowns of the three materials, record baseline and follow-up digital scans of opposing natural teeth at 6 and 12 months, superimpose the scans using the Exocad software to quantify linear changes, compare the amount of wear in the antagonistic tooth, and statistically analyze the differences.

## Materials and methods

Study design and setting

This in vivo comparative clinical study was conducted in the Department of Prosthodontics and Crown and Bridge, Government Dental College and Hospital, Srinagar, India, between October 2024 and December 2025. This study followed a parallel-group design with three arms to evaluate antagonistic tooth wear produced by three different crown materials over a 12-month period. Ethical clearance was obtained from the Institutional Ethical Committee of Government Dental College and Hospital, Srinagar (GDC/Perio/Eth' Committee/1764; dated July 31, 2024). Written informed consent was obtained from all participants after providing a detailed explanation of the procedures, potential risks, and benefits, in their preferred language.

Participants

The patients were recruited from the outpatient department following inclusion and exclusion criteria (Table 1). Thirty-six patients fulfilling the inclusion criteria were enrolled and assigned to three equal groups of 12 patients each. Group 1 received DMLS crowns, Group 2 received rubellite-layered zirconia crowns, and Group 3 received PEEK crowns.

**Table 1:** Inclusion and exclusion criteria for study participants.

Inclusion criteria	Exclusion criteria
Medically healthy adults	Poor oral hygiene
Root canal-treated maxillary first molars requiring full-coverage crowns	Bruxism or other parafunctional habits
Adequate coronal tooth structure with a minimum height of 4 mm	Temporomandibular disorders
Normal occlusion with Class I molar relationship and no crowding	Periodontal disease
Periodontally healthy abutment and opposing teeth	Periapical pathology
Absence of bone resorption on intraoral periapical radiographs	Reduced coronal tooth structure (<4 mm)
Absence of periapical pathology on intraoral periapical radiographs	Neuromuscular disorders

Sample size determination

A priori sample size calculation was performed using the G*Power software (version 3.1; Heinrich Heine University, Düsseldorf, Germany). The effect size (0.55) was derived from the findings of a relevant previous study on antagonist tooth wear [12]. With α = 0.05, statistical power of 80%, and three groups with repeated measures, the minimum total sample size was determined to be 36 participants (12 per group), providing adequate power while accommodating an anticipated dropout rate of 20%.

Clinical procedures

After obtaining informed consent, diagnostic impressions were made using irreversible hydrocolloid material and diagnostic casts were poured into Type III gypsum. Intraoral periapical radiographs confirmed the suitability of these teeth. Tooth preparation followed standard operative protocols, with adequate occlusal clearance specific to each restorative material. Provisional restorations were placed, and final impressions were recorded using an Upcera intraoral scanner (Upcera Dental, Shenyang, China). Crowns were designed virtually using Exocad DentalCAD software (Exocad GmbH, Darmstadt, Germany).

Crown fabrication

Tooth preparation was performed using a standardized protocol, comprising a circumferential shoulder finish line with rounded internal angles, approximately 1.5-2.0 mm of occlusal reduction, 1.0-1.2 mm of axial reduction, and 6-10° total occlusal convergence. The amount of reduction was modified where necessary according to the minimum thickness requirements of the respective restorative material while maintaining a uniform preparation design across all groups. DMLS crowns were fabricated with cobalt-chromium substructures using additive manufacturing followed by veneering with Inspire MC feldspathic porcelain. Rubellite-layered zirconia crowns were produced by milling zirconia copings from rubber zirconia blocks, sintering, and veneering with IPS e.max Ceram (Ivoclar Vivadent AG, Schaan, Liechtenstein). PEEK crowns were milled from PEEK blanks and veneered using Gradia Plus indirect composite (GC Corp., Tokyo, Japan). All restorations underwent standardized finishing, polishing, and glazing protocols according to the manufacturer’s recommendations to optimize surface characteristics. Crowns were tried in, adjusted for occlusion using articulating paper, polished, and cemented under rubber dam isolation using 3M RelyX Resin Cement (3M Oral Care, St. Paul, MN, USA) or appropriate luting agents, according to material-specific protocols. Occlusal contacts were verified to achieve four-point contact on the opposing mandibular first molar.

Follow-up assessment and wear measurement

**Figure 1:**
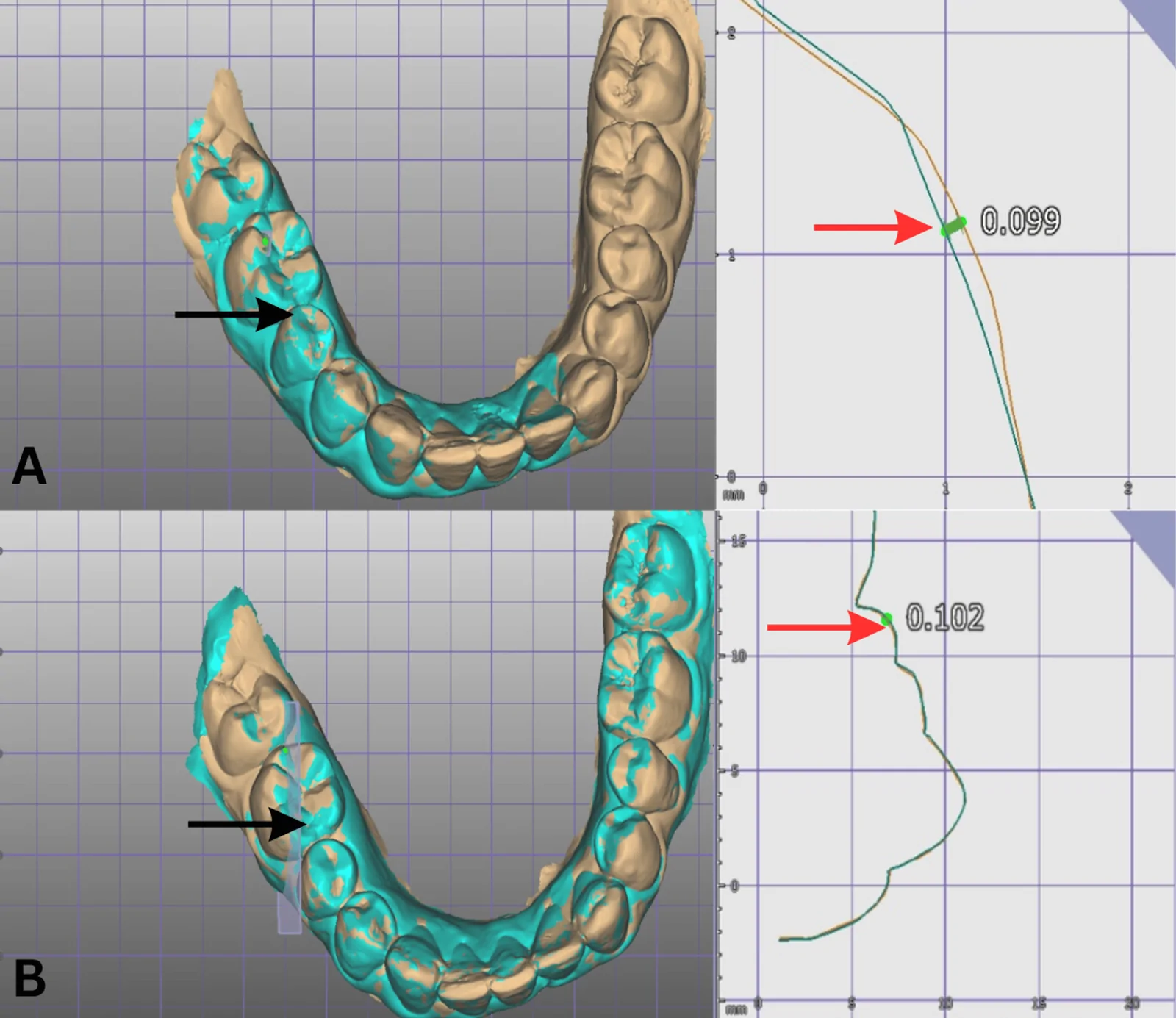
Measurement of linear wear off of anastomosing teeth in mandibular arch by overlapping of scans. (A) At 6th month, green shade area difference (black arrow) and corresponding linear difference (red arrow). (B) At 12th month, green shade area difference (black arrow) and corresponding linear difference (red arrow) follow-up. Images obtained from the ExoCAD software (Exocad GmbH, Darmstadt, Germany).

Baseline digital intraoral scans of the opposing mandibular first molar were obtained 24 hours after cementation using an Upcera intraoral scanner. Patients were recalled at 6 and 12 months for clinical evaluation and repeat scans under standardized conditions (same operator, ambient lighting, and patient positioning). Scans were exported in standard tessellation language (STL) format and superimposed in the Exocad DentalCAD software using best-fit algorithms combined with manual alignment tools where necessary (Figure 1). To reduce measurement bias, linear tooth wear on the occlusal surfaces of the antagonistic mandibular first molars was quantitatively measured by a calibrated examiner blinded to the group allocation.

Statistical analysis

Data were statistically analyzed using IBM SPSS Statistics for Windows, Version 25.0 (Released 2017; IBM Corp., Armonk, NY, USA). Descriptive statistics are expressed as mean ± standard deviation. Normality was verified using the Shapiro-Wilk test. Intragroup analysis was done with a paired t-test for time intervals. Two-way mixed-design analysis of variance (ANOVA) was applied to examine the main effects of material type (DMLS, rubellite-layered zirconia, PEEK) and time (6 and 12 months), along with their interaction, on antagonist tooth wear. When significant main effects were observed, post hoc pairwise comparisons were conducted using Tukey’s Honestly Significant Difference (HSD) test. Statistical significance was set at p < 0.05.

## Results

Antagonistic tooth wear was quantitatively assessed at 6 and 12 months by digital intraoral scanning and surface superimposition. All 36 participants completed follow-up visits, with no dropouts. At the six-month assessment, the mean tooth wear was highest for DMLS crowns, followed by rubellite-layered zirconia crowns and PEEK crowns. A paired t-test was conducted to compare tooth wear values at 6 and 12 months within each crown material group. All three materials demonstrated statistically significant increases in wear from 6 to 12 months (Table 2).

**Table 2:** Comparative analysis of antagonist tooth wear in the study groups at 6 and 12 months with paired t test. Values are presented as mean ± SD in mm, *statistical significance at p < 0.05, effect size (Cohen's d). Data represent antagonist tooth wear measured on the occlusal surface of opposing mandibular first molars using digital scan superimposition. SD: standard deviation; CI: confidence interval; DMLS: direct metal laser sintering; PEEK: polyetheretherketone.

Groups	N (%)	Tooth wear (mm)				Paired t-statistics	p-value	Effect size
		6 months, Mean ± SD	CI at 95%	12 months, Mean ± SD	CI at 95%			
DMLS	12 (33.33)	0.0787 ± 0.0176	0.0675 - 0.0899	0.1018 ± 0.0326	0.0811 - 0.1225	3.89	0.002*	1.12
Rubellite layered zirconia	12 (33.33)	0.0450 ± 0.0238	0.0299 - 0.0601	0.0605 ± 0.0249	0.0447 - 0.0763	4.02	0.001*	1.16
PEEK crown	12 (33.33)	0.0288 ± 0.0123	0.0210 - 0.0366	0.0433 ± 0.0129	0.0351 - 0.0515	7.25	< 0.001*	2.09

A two-way mixed-design ANOVA revealed a statistically significant main effect of crown material type on antagonist tooth wear (F = 38.57, df = 2, p < 0.001, partial η² = 0.539), indicating that the type of restorative material substantially influenced the amount of wear. A significant main effect of time was also observed (F = 11.76, df = 1, p = 0.001, partial η² = 0.151), confirming a progressive increase in wear from 6 to 12 months across all groups. The group × time interaction was not statistically significant (F = 0.28, df = 2, p = 0.759, partial η² = 0.008), suggesting a similar pattern of wear progression over time, irrespective of the material (Table 3).

**Table 3:** Results of two-way mixed-design ANOVA for the effects of group and time on antagonist tooth wear. Two-way mixed-design ANOVA was used for analysis; *statistical significance at p < 0.05. SD: standard deviation; ANOVA: analysis of variance; df: degrees of freedom; η²: eta squared; DMLS: direct metal laser sintering; PEEK: polyetheretherketone.

Source of variation	Sum of squares	df	Mean square	F‑statistic	p‑value	Effect size (Partial η²)
Group (DMLS / Rubellite / PEEK)	0.03698	2	0.01849	38.57	< 0.001*	0.539
Time (6 months vs. 12 months)	0.00564	1	0.00564	11.76	0.001*	0.151
Group × Time (Interaction)	0.00027	2	0.00013	0.28	0.759	0.008

Post hoc pairwise comparisons using Tukey’s HSD test showed that DMLS crowns caused significantly greater antagonist tooth wear than rubellite-layered zirconia crowns (mean difference = 0.0375 mm, p < 0.001) and PEEK crowns (mean difference = 0.0542 mm, p < 0.001). The difference between rubellite-layered zirconia and PEEK crowns was not statistically significant (mean difference = 0.0167 mm, p = 0.130) (Table 4). In summary, DMLS crowns produced the highest antagonistic tooth wear during both evaluation periods, whereas PEEK crowns demonstrated the lowest wear values throughout the study. All the materials showed a time-dependent increase in wear, with PEEK maintaining the most favorable wear behavior in absolute terms.

**Table 4:** Post hoc pairwise comparisons using Tukey HSD test for the main effect of group. * Statistical significance at p < 0.05. HSD: honestly significant difference; CI: confidence interval; DMLS: direct metal laser sintering; PEEK: polyetheretherketone.

Pairwise group comparison	Mean difference (mm)	Standard error	q‑statistic	p‑value	95% CI (lower - upper)
DMLS vs. Rubellite layered zirconia	0.03750	0.00632	5.93	< 0.001*	0.0221 - 0.0529
DMLS vs. PEEK crown	0.05420	0.00632	8.57	< 0.001*	0.0388 - 0.0696
Rubellite layered zirconia vs. PEEK crown	0.01670	0.00632	2.64	0.130	0.0013 - 0.0321

## Discussion

The present in vivo study demonstrated that antagonist tooth wear varied significantly among the three crown materials over 12 months. DMLS crowns produced the highest mean wear at both 6 and 12 months, followed by rubellite-layered zirconia crowns and PEEK crowns, which exhibited the lowest wear values. Two-way mixed-design ANOVA confirmed a strong main effect of material type, a progressive increase in wear over time, and no significant interaction, indicating a consistent relative performance across the observation period. These findings align with the understanding that surface characteristics and veneering materials play a dominant role in abrasive potential beyond the bulk hardness alone.

Layered zirconia was selected because it remains a widely used esthetic restoration, and its veneering ceramic has wear characteristics different from those of monolithic zirconia. The three materials (DMLS, layered zirconia, and PEEK) were chosen to represent metal-ceramic, ceramic, and high-performance polymer restorations with distinct mechanical and tribological properties. Although PEEK is less commonly used than zirconia, its clinical use is increasing because of its favorable biomechanical properties; however, we acknowledge that long-term clinical evidence remains limited. The maxillary first molar was selected because it is frequently restored, experiences high occlusal loads, and provides a standardized antagonist relationship. Only root canal-treated teeth were included to standardize the indication for full-coverage crowns and minimize variability in tooth structure and occlusal conditions across the study groups.

The higher wear associated with DMLS crowns is primarily attributable to feldspathic porcelain veneering (Inspire MC), which contains larger leucite crystals that become exposed after glaze loss during function, leading to increased surface roughness and friction [13]. This is consistent with earlier studies showing that metal-ceramic restorations with conventional feldspathic veneers cause greater enamel abrasion than monolithic or alternative materials [5,13]. In contrast, rubellite-layered zirconia, with its specialized veneering using IPS e.max Ceram (a nanofluorapatite glass ceramic), demonstrated moderate wear [14]. The improved polish retention and smaller crystal size of IPS e.max Ceram likely contributed to its better performance than traditional feldspathic porcelains, supporting reports that lithium disilicate-based veneers exhibit wear closer to that of natural enamel [14].

Recent systematic reviews have reported greater antagonist enamel wear with monolithic zirconia and lithium disilicate restorations than with natural enamel contact [3,4,15]. In contrast, the present study demonstrated that rubellite layered zirconia crowns, utilizing a specialized nano-fluorapatite veneering ceramic, produced significantly less wear than DMLS crowns with conventional feldspathic porcelain. While the findings of Rosa et al. [16] align with the higher abrasive potential observed with feldspathic veneering in our DMLS group, they did not evaluate polymer-based materials, such as PEEK, which showed the most favorable wear behavior in the current investigation. These differences underscore the critical influence of the veneering material composition, surface microstructure, and finishing protocols on clinical wear outcomes.

PEEK crowns veneered with the Gradia Plus indirect composite showed the least antagonist wear throughout the study. This can be explained by the material’s low elastic modulus (closer to dentin), shock-absorbing properties, and capacity of the resin composite for plastic deformation rather than brittle fracture. These attributes reduce stress transmission and abrasive interaction with the opposing enamel [8,17]. These results corroborate previous investigations by Stawarczyk et al. [18] and Kimura et al. [19], which highlighted PEEK’s favorable tribological behavior and minimal enamel wear compared to ceramics and metals. The greater percentage increase in wear observed in the PEEK group from 6 to 12 months, despite the lowest absolute values, may reflect the initial surface adaptation of the composite veneer, yet the overall preservation of enamel remained superior.

Preis et al. [20] demonstrated that zirconia ceramics exhibited superior wear behavior and caused significantly lower antagonistic enamel wear than veneering porcelains. The authors reported that polished or repolished zirconia surfaces produced minimal wear on the opposing enamel, whereas ground or glazed porcelains resulted in a higher abrasive potential. In the present study, the relatively lower wear observed with rubellite-layered zirconia compared to DMLS crowns with feldspathic porcelain veneering further supports the importance of the veneering material type and surface finishing protocols. Grinding procedures have been shown to increase the surface roughness and wear of both ceramics and antagonists, reinforcing the clinical recommendation for meticulous polishing after occlusal adjustment. The digital workflow using intraoral scanning and exocad superimposition proved reliable for quantitative wear assessment, offering advantages in terms of precision and reproducibility over conventional methods. This supports the growing adoption of digital tools in prosthodontic research for longitudinal monitoring [21-23].

Clinically, these results suggest that PEEK crowns may be preferred when preservation of opposing natural dentition is a priority, such as in patients with compromised enamel or parafunctional tendencies (within occlusal guard protection). Rubellite-layered zirconia offers a good balance of aesthetics and acceptable wear behavior for most routine cases, while DMLS crowns should be used judiciously with careful occlusal management and regular monitoring. Proper surface finishing and polishing remain critical, regardless of the material choice, to minimize the long-term abrasive potential.

Limitations of the study include the relatively small sample size and 12-month follow-up period, which may limit the generalizability of the findings and may not fully reflect long-term clinical wear patterns. The study did not directly assess enamel surface roughness, which could provide additional insight into wear mechanisms. Although participants with bruxism and other parafunctional habits were excluded, bruxism severity was not quantitatively evaluated, and dietary habits that may influence enamel wear were not assessed. Furthermore, the study was restricted to single posterior restorations in patients with normal occlusion, limiting extrapolation of the findings to other clinical situations. Future multicenter studies with larger sample sizes, longer follow-up periods, comprehensive assessment of occlusal and dietary factors, and surface characterization analyses are recommended.

## Conclusions

This clinical study demonstrated differences in antagonist enamel wear among the evaluated crown systems over a 12-month period. The observed wear behavior reflects the combined influence of the framework material, veneering material, and surface finishing protocols rather than the framework material alone. Within the limitations of this study, PEEK crown systems exhibited the most favorable enamel-preserving behavior, followed by layered zirconia and DMLS crown systems. Digital intraoral scanning proved to be a precise and noninvasive method for monitoring antagonist tooth wear. Further long-term clinical studies are required to validate these findings across different restorative systems and clinical conditions.
